# Production of Polyhydroxyalkanoates (PHA) by *Haloferax mediterranei* from Food Waste Derived Nutrients for Biodegradable Plastic Applications

**DOI:** 10.4014/jmb.2008.08057

**Published:** 2020-11-17

**Authors:** Ke Wang, Ruihong Zhang

**Affiliations:** Biological and Agricultural Engineering Department, University of California Davis, One Shields Avenue, Davis, CA 95616, USA

**Keywords:** Polyhydroxyalkanoates, food waste, short-chain carboxylates, *Haloferax mediterranei*

## Abstract

Polyhydroxyalkanoates (PHA) are a family of microbial polyesters that are used as biodegradable plastics in replacement of conventional plastics for various applications. However, the high production cost is the barrier for PHA market expansion. This study aimed to utilize food waste as low-cost feedstock to produce poly(3-hydroxybutyrate-co-3-hydroxyvalerate) (PHBV) by *Haloferax mediterranei*. The effects of acetate (Ac), propionate (Pr), butyrate (Bu), and the short-chain carboxylates derived from food waste were examined on the microbial growth and PHBV production. Results showed that a mixture of carboxylates provided a 55% higher PHBV yield than glucose. The food-waste-derived nutrients achieved the yields of 0.41 to 0.54 g PHBV/g Ac from initial loadings of 450 mg/l to 1,800 mg/l Ac of total carboxylates. And the consumption of individual carboxylate varied between different compositions of the carbon source. The present study demonstrates the potential of using food waste as feedstock to produce PHBV by *Haloferax mediterranei*, which can provide economic benefits to the current PHA industry. Meanwhile, it will also help promote organic waste reduction in landfills and waste management in general.

## Introduction

Plastic products are important commodities, with a global production scale of approximately 370 million tons per year, of which 99% are derived from fossil carbon feedstocks [1]. The use of petroleum feedstock for the manufacturing of plastics results in significant emissions of greenhouse gases [2]. Moreover, around 275 million tons of global plastic wastes are generated annually, of which up to 55% end up in landfills or natural environment [3]. U.S. landfills received 26 million tons of plastics wastes, which account for 19% of all landfilled wastes [4]. These environmental issues of conventional plastics urge people to find sustainable alternatives.

Known for the renewable origin and naturally decomposable property, biodegradable plastics are an emerging substitute for conventional ones. As one major type of biodegradable plastics, PHA is a family of high-value polyesters with thermoplastic properties and is used for the manufacturing of various products, *e.g.* packaging films and containers [5]. PHBV is one of the three major types of commercially available PHA, with lower melting temperature, higher elasticity, lower stiffness, higher elongation to break, and increased toughness than the homopolymer Poly(3-hydroxybutyrate) (PHB) [6]. The PHA market currently accounts for 3.3% of global biodegradable plastic market [1], and is projected to reach $0.72 billion by 2020 [7]. One of the current challenges of PHA industry is high production cost largely due to feedstock cost. The production cost of PHA is from $5.0 to 6.1/kg [8], and the feedstock cost accounts for over 40% of the total annual operating cost of PHA production [9]. To reduce feedstock cost, there are many research interests on using inexpensive and waste feedstocks for PHA production.

As intracellular polymers of cells, PHA is produced through microbial cultivation by pure strain or mixed microbial consortium. Pure culture biotechnology using freshwater microbes are commonly seen in industrial scale productions [10], where sterile operation conditions are employed in a production facility [11]. The extreme halophiles, on the contrary, can utilize nonsterile feedstocks without the concern of contamination. *Haloferax mediterranei* is a halophilic archaeon capable of synthesizing PHBV from inexpensive carbon sources [8]. The high salinity facilitates the microbes to grow and synthesize PHBV in unsterile environment with minimum microbial contamination, which can reduce the cost of pasteurization in commercial production. An additional advantage is the simpler extraction process facilitated by the osmotic shock, which may require less energy and chemicals than current production systems.

The majority of recent studies have been using sugar-based substrates to produce PHBV by *H. mediterranei*: hydrolyzed whey [12], extruded rice and corn starch [11], ethanol stillage [13], high-salinity cheese whey hydrolysate [14], molasses wastewater [15] and macroalgal hydrolysates [16] etc. However, *H. mediterranei* excretes extracellular polymeric substances (EPS) when fed with sugar substrates [12, 17], which diverts part of carbon-flow from PHA synthesis [18]. Short-chain carboxylates are essential carbon sources of cell metabolism and direct precursors of PHA synthesis, which could potentially be the better substrate than sugars. Pure volatile fatty acids (VFAs) can be utilized as the sole carbon source by *H. mediterranei* [19]. The strain was shown to grow and synthesize PHA in olive mill wastewater containing VFAs [20].

Food waste is a potential feedstock for PHA production because large quantities of carboxylates can be generated through anaerobic fermentation process, where the production of carboxylates can also be controlled and optimized [21]. At present, there are 6 million tons of food waste generated annually in California, which accounts for 18% of landfill waste. The landfilled food waste is a major source of methane emissions. The utilization of food waste for PHA production will help reduce organic waste in landfills, and reduce feedstock cost due to the current tipping fees associated with the waste disposal.

The objectives of this research were: (1) to understand the effects of various VFAs as the substrate for the cell growth and PHA production by *H. mediterranei* and (2) to examine the production of PHA using food waste as feedstock through a two-stage process. Food waste was firstly degraded into carboxylate-rich nutrients through anaerobic fermentation; then the nutrients were utilized by *H. mediterranei* to produce PHBV in the controlled aerobic process. The results of this study can demonstrate the feasibility of using food waste as feedstock for PHA production. The two-stage bioconversion process could be potentially employed as an innovative and more environmentally friendly approach in the PHA industry.

## Materials and Methods

### Culture Medium and Inoculum Preparation

The wild-type strain *H. mediterranei* (ATCC 33500) was used throughout the study. The cells were firstly cultured in ATCC 1176 medium [22] at 37°C. At the late exponential growth phase, the cell were harvested by using centrifugation and resuspended in minimum salt medium (MSM, containing NaCl, 156 g/l; MgCl_2_.6H_2_O, 13 g/l; MgSO_4_.7H_2_O, 20 g/l; CaCl_2_.6H_2_O, 1 g/l; KCl, 4 g/l; NaBr, 0.5 g/l and FeCl_3_, 5 mg/l) with trace metal solution (SL-6, containing ZnSO_4_.7H_2_O, 100 mg/l; MnCl_2_.4H_2_O, 30 mg/l; H_3_BO_3_, 300 mg/l; C°Cl_2_.6H_2_O, 200 mg/l; CuCl_2_.2H_2_O, 10 mg/l; NiCl_2_.6H_2_0, 20 mg/l; Na_2_MoO_4_.H_2_O, 30 mg/l) [19, 23] and used as inoculum for the following PHA production experiments.

### PHA Production Using Pure VFA and Glucose

Two types of pure carbon sources, VFA mixture (containing acetic acid, propionic acid, and butyric acid with a molar ratio of 2:1.33:1) and glucose were used respectively to give the same initial carbon concentration of 4 g/l. NH_4_Cl was added at 3.06 g/l to give a C/N ratio around 5, which provided excessive nitrogen [18]. KH_2_PO_4_ was added at 0.5 g/l to obtain a C/P ratio about 35, which supplied sufficient phosphorous [19]. MSM and SL-6 were used as the base saline medium. 10 M NaOH was used to adjust the initial pH of both mediums to 7. Bioreactors with 800-ml working volume were used for the aerobic cultivation. The strain was cultured at 37°C with 100 ml/min aeration. Prior to entering the bioreactors, the air flow passed through a humidifier to become moist to prevent water evaporation loss during aeration. The strain was cultured for 144 h until the growth reached stationary phase.

### PHA Production Using Carboxylate-Rich Nutrients Derived from Food Waste

A simulated food waste mixture containing (% w.b.) 28% cooked rice, 32% cooked ground beef and 40%chopped raw cabbage was firstly blended into a slurry using a food processor. The food waste slurry was then anaerobically fermented in 1-L batch reactors with an organic loading of 32 g volatile solids per liter (VS/L) and a food to microorganism (F/M) ratio of 8. The headspace was purged with argon gas to remove air prior to the start of anaerobic fermentation. All the reactors were housed in an incubator controlled at 37°C and mixed twice per day. The pH of fermentation broth naturally dropped from 6 to 5 and remained around 5 afterwards. The fermentation lasted for three weeks until the concentrations of carboxylates were stabilized. After that, the broth was centrifuged at 5,000 rpm for 30 min; the supernatant was then filtered by using 0.2 μm membrane to obtain a particle-free nutrient solution.

The nutrient solution, with carboxylates contents as shown in Table 1, was used as the sole carbon source to culture *H. mediterranei*. The nutrient solution was loaded with four levels: 1.06, 2.12, 3.18, and 4.24 g soluble chemical oxygen demand per liter (sCOD/L), with corresponding contents of total carboxylates as 450, 900, 1,350, and 1,800 mg Ac/l. The base saline medium containing MSM and SL-6, along with sufficient N and P nutrients loadings same to that used in previous experiments. 30 mM NaHCO_3_ was used as buffer to maintain pH at 7.0 for the entire culturing period. The 250-ml bioreactors with 200-ml working volume were used for PHA production. The strain was cultured at 37°C with 100 ml/min aeration for 144 h until the growth reached stationary phase.

### Determination of Cell Mass and Growth Kinetics

To monitor cell growth status, 2 ml of cell broth was collected at different time points during cultivation period and centrifuged at 10,000 rpm for 10 min; the cell mass was resuspended in MSM solution, and the optical density (OD) was analysed at 520 nm [24]. The cell growth rate was obtained as the slope of Ln (OD) versus time. The cell dry mass (CDM) was determined as the volatile suspended solids (VSS) of cell broth: 20 ml of cell broth was sampled and subjected to centrifugation at 8,000 rpm for 20 min. The cell mass was washed with MSM solution twice and subjected to VSS measurement [25]. The cell growth kinetics were curve-fitted based on the Monod model [26]:

$$\mu =\frac{\mu_{m}S}{K_{S}+S}$$

Where, μ is specific growth rate (h^-1^); µ_m_ is maximum specific growth rate (h^-1^); S is total carboxylate loading (g Ac/l); Ks is the half velocity constant (g/l).

### Recovery and Quantification of PHA Production

The strain samples were subjected to transmission electron microscopy (TEM) analysis to observe the cell interiors following a previous method [27]. The extraction of PHA was achieved following a patented method [28] with modifications: the 20 ml cell broth was sampled and centrifuged at 8,000 rpm, 4°C for 20 min. The cell mass was washed with 0.1% sodium dodecyl sulfate (SDS) solution and deionized water. The washed PHA polymers were dried in an oven at 105°C for at least 4 h and the dry weight was measured according to a standard method of total solids (TS) [25]. The extracted PHA was dissolved in 2 ml dichloromethane and 2 ml acidic methanol (3% v/v H_2_SO_4_), with 1 g/l benzoic acid as internal standard. The 12-ml KIMAX culture tubes with rubber lined caps were used for this preparation. The tubes with solution were placed in Hach DRB200 digital reactor block at 105°C for 4 h and cooled to room temperature. The solution was mixed with 1 ml deionized water and settled until phase separation was observed. The organic phase was then transferred to a clean vial for quantification. PHBV with 12% hydroxyvalerate (HV) (Sigma-Aldrich) was used as the standard PHBV chemical. The samples were analysed using a gas chromatograph (GC) method developed based on the methods reported in the literature [29-31] with modifications. The GC (Agilent 6890N) equipped with a flame ionization detector (FID) was employed and the HP-5 capillary column (Agilent, 2016) was used with helium as carrier gas. Details of this method were: inlet temperature and pressure: 230°C, 16 psi; total flowrate: 30 ml/min; split ratio: 8:1; oven temperature: initial 100°C for 2 min; ramping from 100 to 124°C, with a rate of 8°C/min; holding 124°C for 1 min. FID temperature was 240°C, with 40 ml/min of H_2_ flow and 450 ml/min of air flow.

Based on the measured results, the PHBV yield was calculated as the mass of PHBV produced divided by the mass of substrate consumed. Two types of substrate were used in the calculations: glucose and total carboxylates (g Ac/l).

$$Y_{\mathrm{PHBV}}=\frac{{\mathrm{\Delta m}}_{\mathrm{PHBV}}}{{\mathrm{\Delta m}}_{\mathrm{carbon}\mathrm{source}}}$$

The PHBV content of cell was calculated as the percentage of PHBV accounts for the total CDM, described as g VSS/L:

$$\mathrm{PHBV}\%=\frac{m_{\mathrm{PHBV}}}{m_{\mathrm{CDM}}}*100\%$$

The percentage of HV unit in PHBV polymer, which indicates the elasticity of the plastic material, was determined based on the mass ratio between HV unit and the sum of HV and HB units as measured by the GC method described previously:

$$\mathrm{HV}(\%)=\frac{m_{\mathrm{HV}}}{m_{\mathrm{HB}}+m_{\mathrm{HV}}}*100\%$$

### Measurements of Short-Chain Carboxylates and Glucose

To quantify the consumption of substrates, 2 ml of cell broth was collected at different time points during cultivation and centrifuged at 10,000 rpm for 10 min; the supernatant was then filtered through a 0.22 μm membrane. The filtrate was measured for the contents of short-chain carboxylates and glucose by an analytical method described previously [32].

## Results and Discussion

### Intracellular PHBV Generation by *H. mediterranei*

**Fig. 1:**
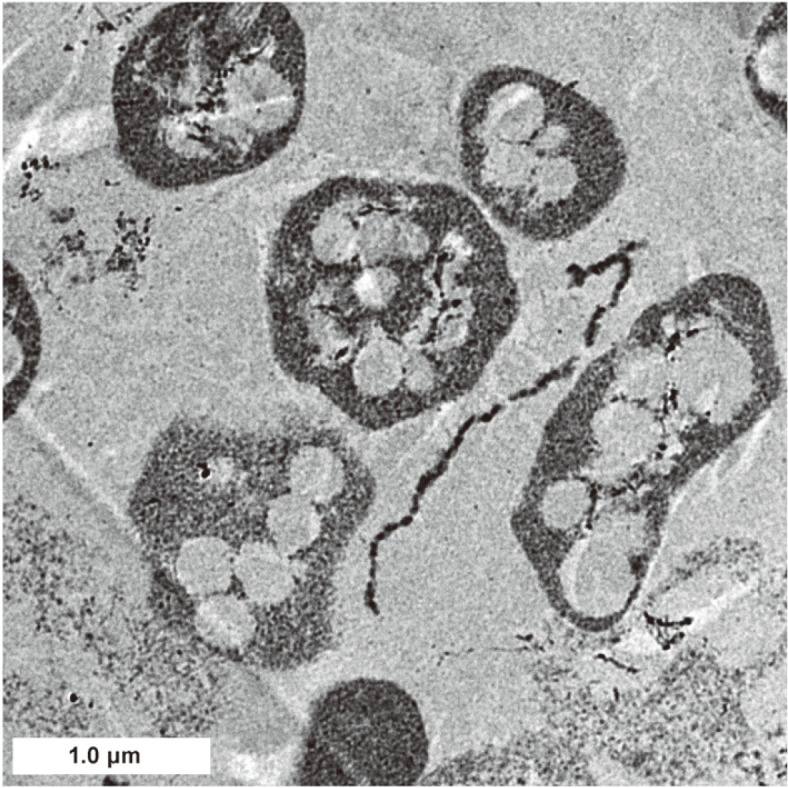
TEM image of *H. mediterranei* cells. The active cells of *H. mediterranei* harvested at late exponential growth phase were subjected to TEM observation immediately, and the oval-shape inclusions in each cell were the accumulated PHBV polymers along with cell growth.

**Fig. 2:**
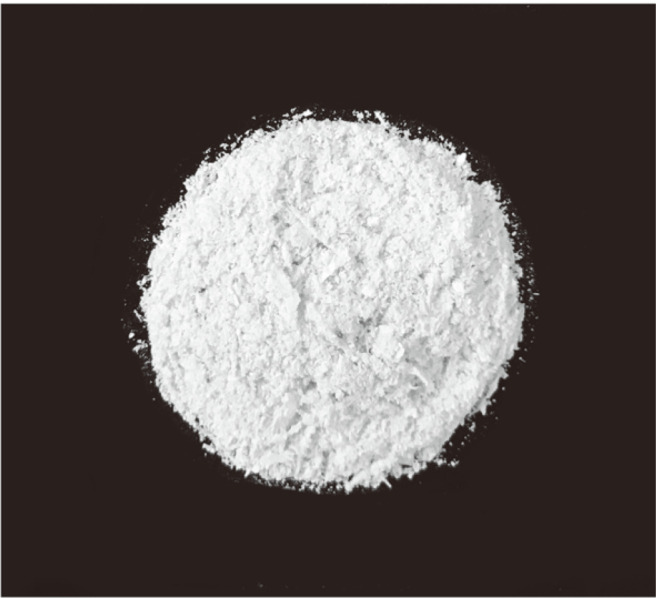
Dried PHBV samples extracted from *H. mediterranei* cells. The PHBV polymers were extracted from the cells of *H. mediterranei* and then washed and dried for storage. The PHBV samples were whitish brittle pieces after oven drying.

*H. mediterranei* has a pinkish appearance formed by a group of red carotenoids which naturally exist in the cell membrane of the extreme haloarchaea [23, 33]. During cell cultivation, the pink color was observed on the strain colonies and cell broth, which indicated normal growth of the species. The species is capable of forming PHBV under normal growth conditions [18]. The PHBV granules with round and oval shapes in the cell interior were captured by TEM, which confirmed the presence of PHBV inclusions in the species (Fig. 1). The dried raw PHBV was the brittle whitish pieces, which can be easily mashed to powder (Fig. 2).

### Pure VFA and Glucose as Sole Carbon Source for PHBV Production

**Fig. 3:**
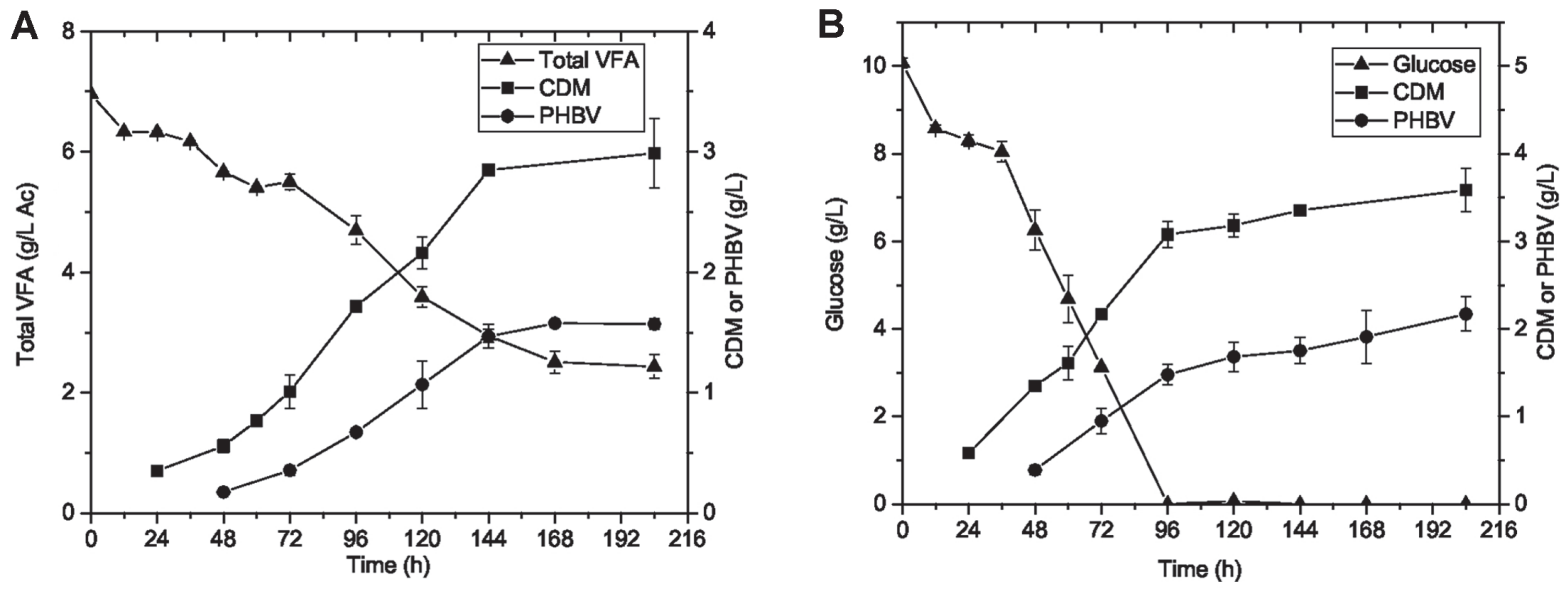
Time course monitoring of PHBV production by *H. mediterranei* by using different carbon sources. VFA mixture including Ac, Pr, and Bu (**A**) and glucose (**B**) were used as the sole carbon source respectively, with the same initial carbon loading of 4 g/l. The production of cell dry mass (CDM) and PHBV and the consumption of each sole carbon source were examined during the cell cultivation period of 204 h.

**Influence of carbon sources on cell growth and PHBV production.** As described in section 2.2, two different types of substrates, pure VFA mixture and glucose, were used individually as the sole carbon source with the same initial carbon concentration of 4 g/l. The maximum concentrations of cell mass and PHBV obtained from pure VFA mixture were 2.99 ± 0.29 g/l and 1.57 ± 0.05 g/l, respectively. In comparison, glucose as carbon source gave slightly higher results: 3.59 ± 0.25 g/l of cell mass and 2.17 ± 0.20 g/l of PHBV as shown in Fig. 3. The growth rate of the species in glucose was 0.032 ± 0.0007 h^-1^; whereas the growth rate in VFA mixture was 0.024 ± 0.0007 h^-1^. A previous study has reported the μ of *H. mediterranei* by using one or two VFA species as carbon source to be 0.010 to 0.035 h^-1^ [19], so the growth rate in VFA mixture obtained in this study was within the reported range. Corresponding to specific growth rate, it took around 96 h for cells to reach stationary phase in glucose, which was shorter than 144 h of cultivation for cells grown in VFA mixture.

The time course monitoring of PHBV production and substrate consumption is shown in Fig. 3. As pure VFA mixture and glucose were consumed respectively as the sole carbon source, PHBV was generated along with cell growth; and a rapid increase of PHBV was observed at the exponential growth phase; the production of PHBV started to decelerate when cell mass was approaching the maximum, and the PHBV concentration remained stable at the stationary phase. For both cases, the PHBV production curve had a very similar trend to the cell growth curve, which suggests the PHBV production was a growth-associated reaction. Similar phenomenon has been observed in previous literature with dominant use of sugar-based feedstocks, *e.g.* glucose, starch and microalgae hydrolysates [16, 18, 24]. It is the first time that the growth-associated PHBV production was observed when short-chain carboxylates were used as the sole carbon source by *H. mediterranei*. In addition to sugar-based carbon sources, the species can also accumulate PHBV by consuming VFA under normal growth conditions. This can be an advantage over some other PHA producers which require necessary growth-limiting factors to stimulate PHA accumulation. This could also be beneficial to large-scale industrial production since PHBV production will not be limited by cell mass in this case.

Particularly when VFA mixture were used as carbon source, the curve of PHBV generation was mostly parallel to that of cell mass production during the entire culturing period. It indicates that the PHBV content of cell dry mass also remained approximately constant when carboxylic nutrients were consumed. The constant PHBV content may be due to the sufficient supply of nutrients and the preferable C/N ratio that maintained the normal growth of *H. mediterranei* during cell cultivation. Based on the current results, it would also be worthwhile to investigate the PHBV production under limited growth conditions, to see if that can lead to more PHA accumulation.

**Fig. 4:**
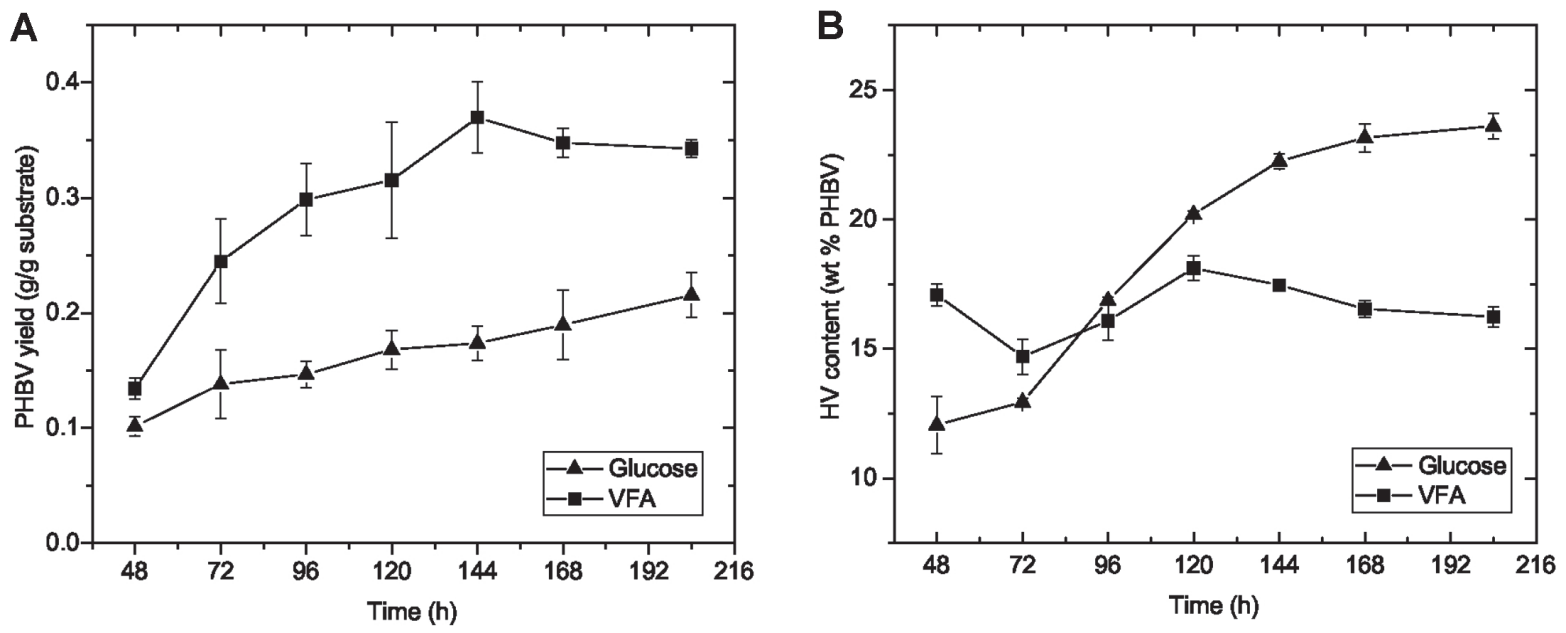
Comparison of PHBV yield and HV content of PHBV polymers by using different carbon sources. Time course PHBV yields by *H. mediterranei* from glucose (g/g glucose) and from VFA mixture (g/g Ac) were examined for the cell cultivation period of 204 h (**A**); Time course HV content of the PHBV polymers produced from glucose and from VFA mixture were compared for the cell cultivation period of 204 h (**B**).

**The influence of carbon source on PHBV yield and HV content.** As shown in Fig. 4, the PHBV yield was calculated based on the mass of PHBV produced and carbon source consumed at each time point during the cultivation period. The yields were around 0.10 g PHBV/g glucose and 0.13 g PHBV/g Ac at 48 h for both carbon sources and increased with strain growth and became stable at the stationary growth phase. The final PHBV yield from pure VFA mixture was higher than that from glucose: 0.34 ± 0.01 g PHBV/g Ac and 0.22 ± 0.02 g PHBV/g glucose.

The higher PHBV yield from VFA than glucose may be due to short-chain carboxylates are the more direct precursors for the synthesis of PHBV than glucose. Short-chain carboxylates (C2 to C5) can be utilized as the sole carbon source for the polymerization of PHBV [19]. In the pathways from carboxylates to PHBV, most carbon flows are towards the polymer formation, except for those used for energy production and CO_2_ loss in tricarboxylic acid (TCA) cycle. Meanwhile the metabolic pathways from glucose to PHBV involve more reaction steps than that from carboxylates [34], which reduces the overall selectivity of PHBV product [35]. Another reason for the lower PHBV yield from glucose is the existence of EPS as a by-product, which also diverts carbon flows from those towards PHBV generation [18].

Carbon source did influence the HV content of PHBV during cultivation process. Under the same carbon loading, the HV content using pure VFA mixture remained stable between 15% to 18% of PHBV during the entire culturing period; meanwhile the HV content using glucose increased from approx. 12% to 24% of PHBV. This may be due to that glucose can be metabolized into HV precursor with the same molar concentration, while only Pr, accounting for part of VFA mixture, was the HV precursor. Since a higher HV content results in more elasticity of the plastic material, it would be feasible to direct the HV content through manipulating the VFA composition for future research interest.

**Fig. 5:**
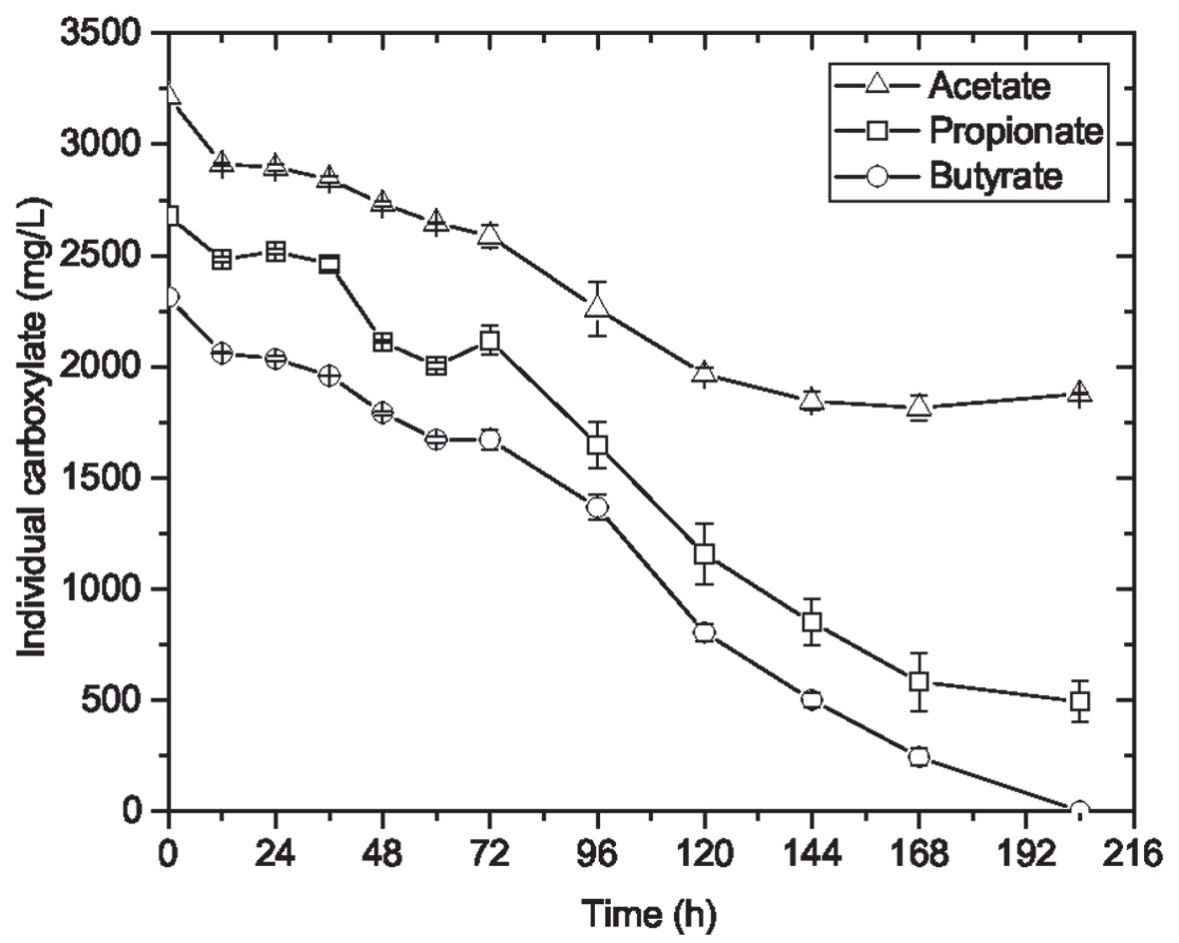
The consumption of individual carboxylate by *H. mediterranei* when using VFA mixture as sole carbon source. The cells of *H. mediterranei* were growing in the VFA mixture including Ac, Pr and Bu with initial carbon loading of 4 g/l; the consumption of each VFA species were analysed during the cell cultivation period of 204 h.

**Substrate consumption**. There was a small portion of VFA leftover when the concentrations of cell mass and PHBV reached maximum and remained stable. As shown in Fig. 5, all three VFA species were consumed during cell growth. However, the extent of the consumption differed among species: by the end of cultivation, Bu was completely consumed, around 81.6% of Pr was consumed, and only 41.6% of Ac was consumed, although all species started with the same mol C% in the mixture. This can be resulted from different substrate uptake rates by *H. mediterranei* for growing and synthesizing PHBV in VFA mixture. The cell growth rate also varies using different VFA species as the single carbon source [19]. The results suggested that a certain composition of the VFA mixture might give better cell growth and PHBV production by *H. mediterranei*, which could be a further research interest.

### PHA Production Using Carboxylate-Rich Nutrients Derived from Food Waste

**Carboxylates and other nutrients from anaerobically fermented food waste.** Around 95% of total carboxylates were obtained after two weeks of anaerobic fermentation of food waste and the production remained almost stable during the week 3. The average total carboxylate yield was 0.35 g/g VS, which was in a reasonable range (0.1 to 0.6 g/g) as previously reported for organic waste [36]. Gases consisting of 38% (v/v) H_2_ and 62% (v/v) CO_2_ were produced and the production of CH_4_ gas was minimum, suggesting the process had been well controlled for acid production with minimum methanogenesis process occurred. The liquid phase of fermented food waste slurry contained over 9 g/l Ac of total carboxylates. The fermentation broth had over 20 g sCOD/L, of which about 76%was from carboxylates.

The soluble nutrient composition of the fermentation liquid is shown in Table 1. The carboxylates produced from food waste contained approximately 35% Ac, 13% Pr and 46% Bu as the dominant species. This compositional result agreed with previous literature producing carboxylates from different types of food waste through acidogenesis: the mesophilic acidogenic culture obtained 28% Ac, 15% Pr and 57% Bu from dining hall food waste [37]; and 32% Ac, 10% Pr and 58% Bu were obtained from heat-treated food waste with pH 5 and mesophilic condition [38]. Although carboxylate composition varies among studies, Ac, Pr, and Bu are the three major species generated from anaerobic fermentation process.

**Fig. 6:**
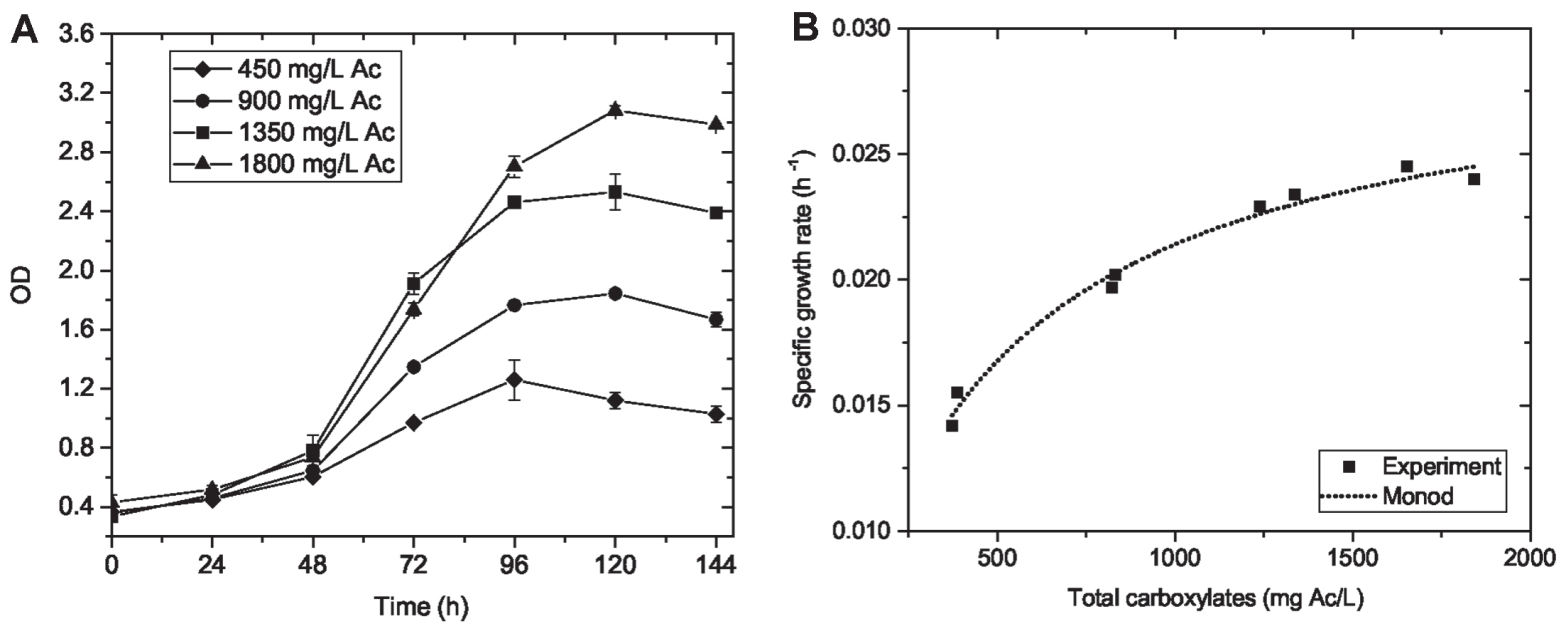
The status of cell growth and growth kinetics of *H. mediterranei* in food waste derived nutrients. Cell growth curve of *H. mediterranei* with different initial loadings of waste-derived carboxylates were examined for the cell cultivation period of 144 h (**A**); the cell growth kinetics on waste-derived carboxylates were examined by using curve-fitting method according to Monod model (with an adjusted R^2^ = 0.983) (**B**).

**The influence of substrate loading on cell growth.** The cell growth curves with different substrate loadings (450, 900, 1,350, and 1,800 mg Ac/l) are illustrated in Fig. 6. As an indicator of cell number, the OD of cell broth in all substrate loadings increased with time and reached a plateau around 120 h after inoculation. At the stationary growth phase, the final OD increased with higher initial substrate loadings, which were also indicated by the increasing extent of pink color of cell broth. This is reasonable since a higher nutrient concentration would be converted to more cell mass, if the substrate loading did not cause any inhibition effect on cell growth. Besides, indicated by the slope of the growth curve, the μ in all substrate loadings started to increase rapidly around 24 h after inoculation and remained mostly exponential growth through 96 h. The μ decreased afterwards, which may be a consequence of nutrient depletion or cell mass loss due to endogenous cell metabolism [39].

Based on the results of OD from 24 h to 96 h when the most rapid growth happened, the μ was calculated to be from 0.014 to 0.025 h^-1^ and increased with higher loadings of total carboxylates. The cell growth rate from waste-derived carboxylic nutrients was very similar to that from pure carboxylic nutrients. As shown in Fig. 6, the results of experimental μ were used to establish the Monod model through curve-fitting, which reflexed the μ as a function of total carboxylate concentration. The cell growth kinetics were also obtained from the Monod model, with an adjusted R^2^ of 0.983. The modelled *µ*_m_ was 0.0296 ± 0.0007 h^-1^; the Ks was 383 ± 34 mg/l. The modelled reaction kinetics suggested higher substrate loadings would be possible to obtain higher specific growth rate, which would be beneficial for the steady-state and other continuous production scales. Previous studies have reported the possible inhibition effect of VFA on cell growth of some PHA-producers at high concentrations [40]; therefore, a further research interest is to investigate possible inhibition effect of high substrate loadings of the waste-derived carboxylic nutrients on the cell growth of *H. mediterranei*.

**Fig. 7:**
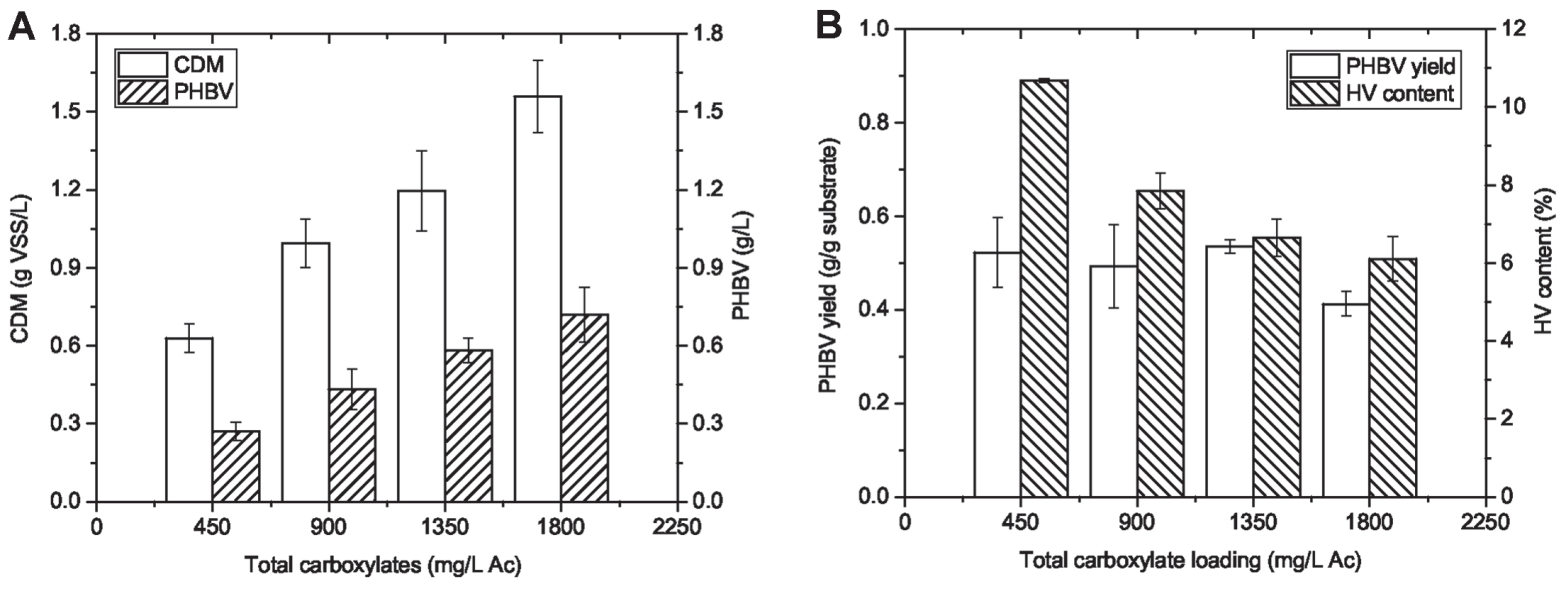
The production and yield of PHBV and the HV content of the polymers produced from food waste derived nutrients. The production of CDM and PHBV by *H. mediterranei* from different loadings of food waste derived carboxylic nutrients were examined at the stationary growth phase (**A**); the PHBV yield and HV content of the polymers produced from different loadings of food waste derived nutrients were examined at 144 h (**B**).

**The influence of substrate loading on PHBV production.** Like cell mass production trend, the concentration of PHBV increased with higher loadings of fermented food waste permeate from 450 to 1,800 mg Ac/l. As shown in Fig. 7, the PHBV content of cell biomass was generally stable among different substrate loadings, which was in the range of 43% to 57% (% CDM). This was the reason for the increasing PHA concentrations with higher cell mass production. Similar results of PHA content in cells have been reported for *H. mediterranei* under balanced growth conditions by other studies, as shown in Table 2. Apart from this strain, very similar results of PHA content in cells have also be obtained by other PHA-producing microorganisms using VFA as carbon source. The PHA content can be higher under unbalanced growth conditions, *e.g.* when nitrogen source is limited and C/N ratio is high [18]. Similar concept of nutrient limitation is employed when feast-and-famine strategies are used for the mixed consortium of microorganism as the PHA-producer [41]. Some genetic modifications can also trigger the cells to accumulate more PHA intracellularly [10, 42].

The PHBV yield remained in the range of 0.41 to 0.54 g PHBV/g Ac, with 450 mg/l to 1,800 mg/l Ac of total carboxylates loadings. The results suggested that short-chain carboxylates, either pure chemicals or derived from food waste fermentate, can be a good option of the carbon source that achieves high PHBV yield by *H. mediterranei*.

The average HV contents from waste-derived carboxylic nutrients were in the range of 6% to 11% and decreased with higher total carboxylates loadings. Based on current literature, glucose, Pr and Va were found to be the precursors of HV units in the PHBV synthesis [19, 34]. The reason for the lower HV content from carboxylic nutrients may be due to the low existence or consumption of Pr and Va. Higher HV content in the PHBV can increase the elasticity of the polymer, which can be desirable for wider biodegradable plastic applications. Therefore, this could be a further research interest to obtain high PHBV yields with a high HV content using organic waste streams rich in carboxylic nutrients.

**Fig. 8:**
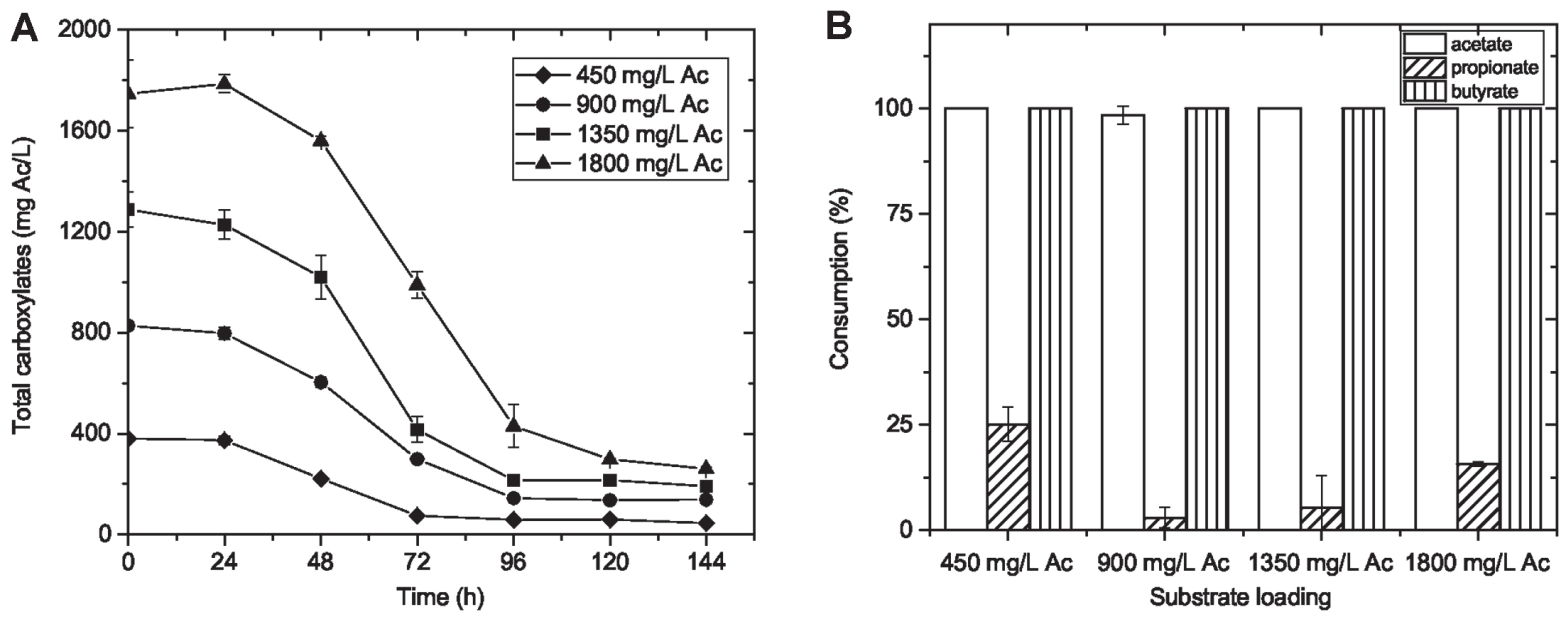
The consumption of short chain carboxylates by *H. mediterranei* with different substrate loadings. The time course consumption of total short chain carboxylates by *H. mediterranei* with different initial substrate loadings were examined for the cell cultivation period of 144 h (**A**); Individual carboxylate consumption by *H. mediterranei* with different initial substrate loadings were analyzed at 144 h (**B**).

**Substrate consumption.** The total carboxylate (as Ac equivalent) consumption with different substrate loadings during the entire culturing period is shown in Fig. 8. The substrate consumption curves of different substrate loadings had a similar trend: the total carboxylates decreased rapidly from 24 to 96 h and remained stable afterwards. At the end of cell growth and PHBV production, 84% to 88% of the total carboxylates were consumed (the initial substrate loadings were 450 to 1,800 mg Ac/l). This was promising for organic reduction in waste streams. In terms of the consumption of individual carboxylate, almost 100% consumption was achieved for Ac, iso-Bu, Bu, and isovalerate (iso-Va) for all substrate loading levels. However, less than 25% of Pr was consumed in all loading levels, which corresponds to the low HV content of the PHA produced from waste-derived permeates. It is worthwhile to notify that most of Pr was consumed and the majority of Ac was left out when pure carboxylates were used in previous experiment. These results indicate that although all species of short-chain carboxylates are feasible nutrients, the strain might have a different uptake preference on each carboxylate species at various substrate conditions, *e.g.* when the composition of carboxylic mixture varies, and other soluble nutrients exist in the medium.

## Figures and Tables

**Table 1:** Composition of nutrients derived from food waste.

Compound	Unit	Average value (standard deviation)
sCOD (total)	mg/L	21170 (1598)
sCOD (from carboxylates)	mg/L	17161 (665)
Total nitrogen	mg/L N	24 (0)
Ammonium	mg/L N	20 (4)
Total phosphorous	mg/L PO_4_^3-^	38 (1)
Ash	mg/L	3720 (85)
Total short-chain carboxylates	mg/L Ac	9136 (255)
Ac	mg/L	3982 (142)
Pr	mg/L	1403 (328)
iso-Bu	mg/L	375 (32)
Bu	mg/L	5150 (568)
Iso-Va	mg/L	236 (41)
Va	mg/L	76 (11)
Lactate (La)	mg/L	103 (18)

**Table 2:** Some literature results of PHA content by *H. mediterranei* and other species.

Substrate	Microbial species	CDM g/l	PHA content % CDM	PHA yield g/g	HV content % PHA	Reference
Olive mill wastewater	*H. mediterranei*	0.47	43	-	6.5	[20]
Synthetic VFA	*H. mediterranei*	1.2-7.7	19.9	0.11	-	[19]
Glucose and Va	*H.mediterranei ES1 (EPS gene deleted)*	0.4-13.3	41	-	9.1-53.3	[43]
Cheese whey hydrolysate	*H. mediterranei*	7.5	53	0.78	1.5	[14]
Extruded corn starch, rice bran	*H. mediterranei*	140.0	55.6	-	-	[11]
Glucose	*H. mediterranei*	1.4-15.8	36	0.86	8.8-12.5	[18]
Synthetic VFA	*H. palleronii*	0.9-1.6	63	0.05	-	[44]
Brewery wastewater	*Activated sludge*	-	39	0.43	-	[45]
Fermented food waste	*H. mediterranei*	0.6-3.0	43-57	0.41-0.54	6.1-10.7	This study

## References

[1] European Bioplastics (2018). Bioplastic Market Data [Internet]. European Bioplastics e.V..

[2] Criddle CS, Frank CW (2014). Renewable bioplastics and biocomposites from biogas methane and waste-derived feedstock: development of enabling technology, life cycle assessment, and analysis of costs.

[3] Geyer R, Jambeck JR, Law KL (2017). Production, use, and fate of all plastics ever made. Sci. Adv..

[4] US EPA (2019). Plastics: Material-Specific Data | Facts and Figures about Materials, Waste and Recycling | US EPA [Internet].

[5] Reddy CSK, Ghai R, Rashmi, Kalia VC (2003). Polyhydroxyalkanoates: an overview. Bioresour. Technol..

[6] Anjum A, Zuber M, Zia KM, Noreen A, Anjum MN, Tabasum S (2016). Microbial production of polyhydroxyalkanoates (PHAs) and its copolymers: a review of recent advancements. Int. J. Biol. Macromol..

[7] Aeschelmann F, Carus M (2015). Biobased building blocks and polymers in the world: capacities, production, and applications-status quo and trends towards 2020. Ind. Biotechnol..

[8] Kourmentza C, Plácido J, Venetsaneas N, Burniol-Figols A, Varrone C, Gavala HN (2017). Reis MAM. recent advances and challenges towards sustainable polyhydroxyalkanoate (PHA) production. Bioengineering.

[9] Leong YK, Show PL, Lan JC-W, Loh H-S, Lam HL, Ling TC (2017). Economic and environmental analysis of PHAs production process. Clean Technol. Environ. Policy.

[10] Chen GQ (2009). A microbial polyhydroxyalkanoates (PHA) based bio- and materials industry. Chem. Soc. Rev..

[11] Huang T-Y, Duan K-J, Huang S-Y, Chen CW (2006). Production of polyhydroxyalkanoates from inexpensive extruded rice bran and starch by *Haloferax mediterranei*. J. Ind. Microbiol. Biotechnol..

[12] Koller M, Hesse P, Bona R, Kutschera C, Atlić A, Braunegg G (2007). Biosynthesis of high quality polyhydroxyalkanoate co- and terpolyesters for potential medical application by the archaeon *Haloferax mediterranei*. Macromol. Symposia.

[13] Bhattacharyya A, Saha J, Haldar S, Bhowmic A, Mukhopadhyay UK, Mukherjee J (2014). Production of poly-3-(hydroxybutyrate-cohydroxyvalerate) by *Haloferax mediterranei* using rice-based ethanol stillage with simultaneous recovery and re-use of medium salts. Extremophiles.

[14] Pais J, Serafim LS, Freitas F, Reis MAM (2016). Conversion of cheese whey into poly(3-hydroxybutyrate-co-3-hydroxyvalerate) by *Haloferax mediterranei*. New Biotechnol..

[15] Cui Y-W, Zhang H-Y, Ji S-Y, Wang Z-W (2017). Kinetic analysis of the temperature effect on polyhydroxyalkanoate production by *Haloferax mediterranei* in synthetic molasses wastewater. J. Polym. Environ..

[16] Ghosh S, Gnaim R, Greiserman S, Fadeev L, Gozin M, Golberg A (2019). Macroalgal biomass subcritical hydrolysates for the production of polyhydroxyalkanoate (PHA) by *Haloferax mediterranei*. Bioresour. Technol..

[17] Parolis H, Parolis LAS, Bo IF, Rodriguez-Valera F, Manta MC, Jansson P-E, Sutherland IW (1996). The structure of the exopolysaccharide produced by the halophilic Archaeon *Haloferax mediterranei* strain R4 (ATCC 33500). Carbohydr. Res..

[18] Cui Y-W, Shi Y-P, Gong X-Y (2017). Effects of C/N in the substrate on the simultaneous production of polyhydroxyalkanoates and extracellular polymeric substances by *Haloferax mediterranei* via kinetic model analysis. RSC Adv..

[19] Ferre-Guell A, Winterburn J (2018). Biosynthesis and characterization of polyhydroxyalkanoates with controlled composition and microstructure. Biomacromolecules.

[20] Alsafadi D, Al-Mashaqbeh O.A (2017). One-stage cultivation process for the production of poly-3-(hydroxybutyrate-cohydroxyvalerate) from olive mill wastewater by *Haloferax mediterranei*. New Biotechnol..

[21] Zhou M, Yan B, Wong JWC, Zhang Y (2018). Enhanced volatile fatty acids production from anaerobic fermentation of food waste: a mini-review focusing on acidogenic metabolic pathways. Bioresour. Technol..

[22] ATCC (2020). *Haloferax mediterranei* (Rodriguez-Valera et al.) Torreblanca et al. AT [Internet].

[23] Dyall-Smith M (2009). The Halohandbook Protocols for halobacterial genetics.

[24] Lillo JG, Rodriguez-Valera F (1990). Effects of culture conditions on Poly(3-Hydroxybutyric Acid) production by *Haloferax mediterranei*. Appl. Environ. Microbiol..

[25] APHA (2012). Standard methods for the examination of water and wastewater.

[26] Monod J (1949). The growth of bacterial cultures. Ann. Rev. Microbiol..

[27] Lu Q, Han J, Zhou L, Zhou J, Xiang H (2008). Genetic and biochemical characterization of the Poly(3-Hydroxybutyrate-co-3-Hydroxyvalerate) synthase in *Haloferax mediterranei*. J. Bacteriol..

[28] Escalona AM, Varela FR, Gomis AM (1996). Procedure for extraction of polyhydroxyalkanoates from halophilic bacteria which contain them [Internet]. US5536419A.

[29] Braunegg G, Sonnleitner B, Lafferty RM (1978). A rapid gas chromatographic method for the determination of poly-?-hydroxybutyric acid in microbial biomass. Eur. J. Appl. Microbiol. Biotechnol..

[30] Lemos PC, Viana C, Salgueiro EN, Ramos AM, Crespo JPSG, Reiszcorr MAM (1998). Effect of carbon source on the formation of polyhydroxyalkanoates (PHA) by a phosphate-accumulating mixed culture. Enzyme Microb. Technol..

[31] Oehmen A, Keller-Lehmann B, Zeng RJ, Yuan Z, Keller J (2005). Optimisation of poly-β-hydroxyalkanoate analysis using gas chromatography for enhanced biological phosphorus removal systems. J. Chromatogr. A.

[32] Sluiter A (2008). Determination of sugars, byproducts, and degradation products in liquid fraction process samples: Laboratory Analytical Procedure (LAP); Issue Date: 12/08/2006. Technical Report.

[33] Fang C-J, Ku K-L, Lee M-H, Su N-W (2010). Influence of nutritive factors on C50 carotenoids production by *Haloferax mediterranei* ATCC 33500 with two-stage cultivation. Bioresour. Technol..

[34] Han J, Hou J, Zhang F, Ai G, Li M, Cai S (2013). Multiple Propionyl Coenzyme A-Supplying Pathways for Production of the Bioplastic Poly(3-Hydroxybutyrate- co -3-Hydroxyvalerate) in *Haloferax mediterranei*. Appl. Environ. Microbiol..

[35] Fogler HS (2016). Elements of chemical reaction engineering.

[36] Arslan D, Steinbusch KJJ, Diels L, Hamelers HVM, Strik DPBTB, Buisman CJN (2016). Selective short-chain carboxylates production: a review of control mechanisms to direct mixed culture fermentations. Crit. Rev. Environ. Sci. Technol..

[37] Shin H-S, Youn J-H, Kim S-H (2004). Hydrogen production from food waste in anaerobic mesophilic and thermophilic acidogenesis. Int. J. Hydrog. Energy.

[38] Kim D-H, Kim S-H, Jung K-W, Kim M-S, Shin H-S (2011). Effect of initial pH independent of operational pH on hydrogen fermentation of food waste. Bioresour. Technol..

[39] Shuler ML, Kargi F (2008). Bioprocess engineering: basic concepts.

[40] Yu J, Si Y, Keung W, Wong R (2002). Kinetics modeling of inhibition and utilization of mixed volatile fatty acids in the formation of polyhydroxyalkanoates by Ralstonia eutropha. Process Biochem..

[41] Korkakaki E, Mulders M, Veeken A, Rozendal R, van Loosdrecht MCM, Kleerebezem R (2016). PHA production from the organic fraction of municipal solid waste (OFMSW): overcoming the inhibitory matrix. Water Res..

[42] Madison LL, Huisman GW (1999). Metabolic engineering of Poly(3-Hydroxyalkanoates): from DNA to plastic. Microbiol. Mol. Biol. Rev..

[43] Han J, Wu L-P, Hou J, Zhao D, Xiang H (2015). Biosynthesis, characterization, and Hemostasis potential of tailor-made Poly(3-hydroxybutyrate- co -3-hydroxyvalerate) produced by *Haloferax mediterranei*. Biomacromolecules.

[44] Venkateswar Reddy M, Mawatari Y, Yajima Y, Satoh K, Venkata Mohan S, Chang Y-C (2016). Production of poly-3-hydroxybutyrate (P3HB) and poly(3-hydroxybutyrate-co-3-hydroxyvalerate) P(3HB-co-3HV) from synthetic wastewater using Hydrogenophaga palleronii. Bioresour. Technol..

[45] Ben M, Kennes C, Veiga MC (2016). Optimization of polyhydroxyalkanoate storage using mixed cultures and brewery wastewater. J. Chem. Technol. Biotechnol..

